# Elastographic Stiffness of the Rotator Cuff Pre and Post Surgical Repair in 115 Consecutive Patients

**DOI:** 10.1177/23259671261451756

**Published:** 2026-07-10

**Authors:** Antonette Mariama R. Bilog, Mina Shenouda, Patrick H. Lam, George A.C. Murrell

**Affiliations:** * Orthopaedic Research Institute, St. George Hospital Campus, University of New South Wales, Sydney, NSW, Australia; Investigation performed at Orthopaedic Research Institute, St. George Hospital Campus, University of New South Wales, Sydney, New South Wales, Australia

**Keywords:** shoulder, rotator cuff, clinical medicine by anatomic region, imaging, diagnostic ultrasound, clinical medicine by specialty interest, biomechanics of tendon, research (in vivo or in vitro), biology of tendon

## Abstract

**Background:**

Higher retear rates after rotator cuff repair have been reported in older patients with large tears. Shear wave elastography (SWE) offers a noninvasive, objective way to assess tendon stiffness, yet the relationship between the preoperative and postoperative elastographic stiffness of the supraspinatus tendon has not been well studied.

**Purpose:**

To determine the association between preoperative and 6-week postoperative supraspinatus tendon elastographic stiffness and to assess the effect of age, symptom duration, tear thickness, and tear size on these measurements.

**Study Design:**

Case series; Level of evidence, 4.

**Methods:**

Included were 115 consecutive shoulders undergoing primary arthroscopic repair of isolated supraspinatus tears using the undersurface technique. SWE measurements were obtained of the torn supraspinatus tendon preoperatively and of the repaired tendon 6 weeks postoperatively using a standardized protocol.

**Results:**

The preoperative elastographic stiffness of the torn edge of the supraspinatus tendon was diminished in larger (*R* = −0.3; *P* < .0001), full-thickness (*R* = −0.4; *P* = .0001) tears and in older patients (*R* = −0.3; *P* = .002). The mean (± standard deviation) SWE velocity of the supraspinatus tendon decreased by 12% 6 weeks postoperatively (6 ± 1.8 m/s preoperatively vs 5 ± 0.85 m/s postoperatively). While a modest correlation existed between preoperative and 6-week postoperative SWE (*R* = 0.3; *P* = .01), a strong inverse relationship was found between preoperative SWE and the improvement in elastographic stiffness after repair (change in SWE [ΔSWE]) (*R* = −0.8; *P* < .0001). Multivariate analysis identified preoperative SWE as the strongest predictor of ΔSWE (*F* = 60; *P* < .0001).

**Conclusion:**

Preoperative elastographic stiffness of the supraspinatus tendon was only modestly associated with early postoperative elastographic stiffness, a finding largely explained by a regression-toward-the-mean effect. These data support the hypothesis that the elastographic stiffness of supraspinatus tendons decreases with advancing age and with larger full-thickness tears. While it was observed that these tendons were elastographically softer preoperatively, they demonstrated the capacity for significant improvement in elastographic stiffness after repair.

Rotator cuff repair is a commonly performed procedure, yet structural failure after repair occurs often at a rate >50% for large and massive tears.^5,14,21^ Increasing age and larger tear size are consistently associated with higher rates of retear.^13,14,21^

Several basic science studies have led to the hypothesis that supraspinatus tendons in older patients with larger tears may have reduced healing potential.^2,15^ Benson et al^2^ obtained fresh rotator cuff tendon samples from 27 patients with varying degrees of subacromial impingement and tears. Histological sections demonstrated increased tissue hypoxia and apoptosis in full-thickness tears. Matthews et al^15^ likewise did biopsies of the rotator cuff tendons of 40 patients with chronic rotator cuff disease. Histological sections exhibited increased reparative and inflammatory changes in smaller partial-thickness tears and decreased cellular activity and vascular proliferation in larger full-thickness tears.

Soft tissue injuries, such as rotator cuff tears and retears, are often evaluated using ultrasound.^3,11,12^ Shear wave elastography (SWE) is a relatively novel quantitative sonoelastographic technique that measures tissue stiffness in vivo.^1,7,9,17,20^ SWE application to the assessment of rotator cuff tendons has emerged only recently.^3,8,10,16^ In our institution, we have found that the postoperative elastographic stiffness of the supraspinatus tendon is lower in older patients and in patients with larger tears.^16^ We observed that the elastographic stiffness of the repaired tendon changes over time, particularly in the first 3 months, with an overall mean increase of 22% from 1 to 52 weeks.^8^

To date, studies using SWE in the evaluation of rotator cuff repairs have focused predominantly on postoperative measurements. The elastographic properties of the torn supraspinatus tendon before surgery, and their relationship to postoperative tendon elastographic stiffness, have not been examined. In a recent study, we demonstrated that preoperative SWE measurements of torn supraspinatus tendons were feasible in tears <3 cm in size, when the tendon edge is not too far retracted medially under the acromion.^19^ The ability to measure the elastographic stiffness of the majority of torn tendons before surgery created an opportunity in this study to directly assess tendon elastographic stiffness before repair and investigate the relationship between preoperative and postoperative SWE measurements of the supraspinatus tendon.

The primary aim of this study was to determine the association, if any, between the preoperative elastographic stiffness of the torn supraspinatus tendon and its elastographic stiffness 6 weeks after arthroscopic primary repair. Secondary aims were to examine the relationships between the preoperative elastographic stiffness of the supraspinatus tendon and age, duration of symptoms, tear thickness, and tear size; and to identify which factors are associated with the change in elastographic stiffness of the tendon after repair.

## Methods

This was a case series (level of evidence, 4) designed to evaluate the relationship between the preoperative and 6-week postoperative elastographic stiffness of the supraspinatus tendon measured using ultrasound SWE. Our analysis focused on the early postoperative period as the 6-week time point corresponds to a clinically important stage of tendon-bone healing and postoperative rehabilitation. The primary outcome was the association between preoperative and 6-week postoperative SWE (velocity [m/s]; elasticity [kPa]) of the supraspinatus tendon. Secondary outcomes were the change in SWE (ΔSWE) from before surgery to 6 weeks after surgery and the association of age, duration of symptoms, tear thickness, tear size, and preoperative SWE with postoperative SWE and ΔSWE.

Consecutive patients undergoing arthroscopic primary repair of a rotator cuff tear with involvement of the supraspinatus tendon and with ultrasound and SWE assessment before surgery and at 6 weeks after surgery were selected. Patients with an isolated subscapularis tear, supraspinatus tendon tear deemed irreparable, previous surgery on the same shoulder (ie, revision rotator cuff repair surgery), surgery involving augmentation or use of a synthetic patch, or rotator cuff calcific tendonitis were excluded.

Ethical approval was obtained from the South Eastern Sydney Local Health District–Human Research HREC in accordance with the National Statement on Ethics Conduct in Human Research 2023 (NHMRC) (HREC Approval 202135, 2019/ETH14049).

### Ultrasound and SWE Assessment

All ultrasound and SWE examinations were performed by a single experienced musculoskeletal sonographer (M.S.) using the ACUSON Sequoia Select Ultrasound System (Siemens Medical Solutions) and a standardized protocol. Patients were seated with the shoulder in neutral rotation, 0° of abduction, and 30° to 35° extension. The elbow was flexed 90° and the forearm supinated and supported on an arm rest. The transducer was placed over the supraspinatus fossa to obtain a longitudinal view of the tendon with the acromion medially and the greater tuberosity laterally. Static B-mode images were captured in the longitudinal plane and used to measure tear thickness, tear size, and SWE.

**Figure 1.**
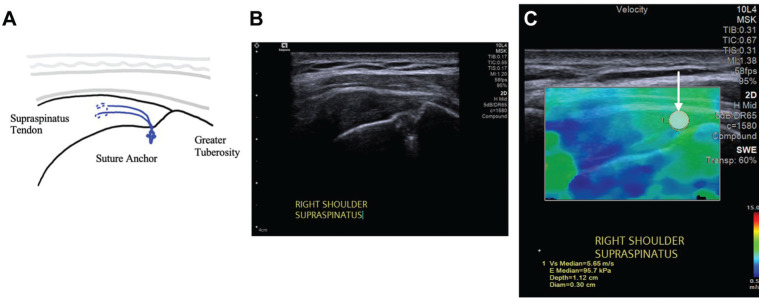
(A) Diagram of the repaired supraspinatus tendon using the undersurface technique with 1 suture anchor. (B) Static B-mode ultrasound grayscale image. Longitudinal view of the repaired supraspinatus tendon at 6 weeks after surgery. (C) Color elastogram. Red indicates higher velocity (m/s) or elasticity (kPa), blue indicates lower velocity (m/s) or elasticity (kPa), and the circle with a white arrow indicates the region of interest placed over the insertion of the repaired tendon into the greater tuberosity.

Preoperatively, SWE measurements were obtained from the medial stump of the torn supraspinatus tendon approximately 6 mm from its edge. The region of interest circle was placed over an area of the tendon with good tissue image, indicated by green on the quality map.^19^ Postoperatively, SWE measurements were taken from the same region of the tendon 6 mm from the bone-tendon repair interface, avoiding any suture material.^8,16,18,19^ SWE was recorded as both velocity (m/s) and elasticity (kPa) (Figure 1). Similar effects were noted for both measures; therefore, the majority of our data are presented in meters per second for clarity.

### Surgical Technique

All surgeries were performed by a single orthopaedic shoulder specialist (G.A.C.M.) using a standardized arthroscopic rotator cuff repair undersurface technique with knotless inverted mattress sutures deployed using the Opus Smart Stitch Suture Device and fixed with Opus Magnum Knotless anchors (Smith & Nephew), as previously described.^4,6,18^ Postoperatively, the operated shoulder was immobilized in an arm sling with an abduction pillow for 6 weeks to relieve tension on the repaired supraspinatus tendon.

### Statistical Analysis

Statistical analysis was performed using IBM SPSS Statistics software. Correlation studies were assessed using the Spearman rho coefficient, with a *P* value <.05 considered statistically significant. Strength of association was defined as 0 to 0.19 very weak, 0.2 to 0.39 weak, 0.4 to 0.59 moderate, 0.6 to 0.79 strong, 0.8 to 1.0 very strong. Univariate and multivariate linear regression analyses (analyses of variance) were used to evaluate predictors of ΔSWE. The Oldham method was used to correct for statistical coupling between preoperative SWE and ΔSWE.

## Results

**Figure 2.**
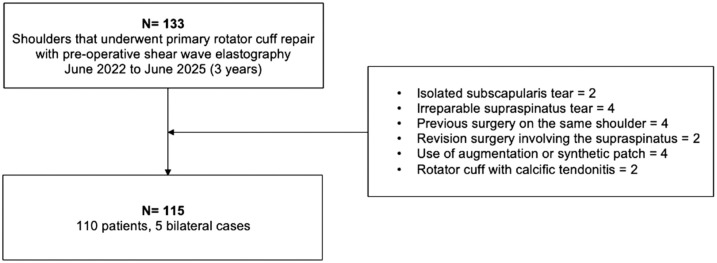
Patient recruitment and final sample size after application of inclusion and exclusion criteria.

A total of 133 consecutive patients underwent arthroscopic primary repair of the supraspinatus tendon and ultrasound testing with SWE before surgery and 6 weeks after surgery. Those with an isolated subscapularis tear, an irreparable supraspinatus tear, previous surgery on the same shoulder, revision surgery involving the supraspinatus, use of augmentation or a synthetic patch, and rotator cuff calcific tendonitis were excluded (n = 18), leaving a final sample of 115 shoulders (110 patients, 5 bilateral) (Figure 2). A sample size of >100 shoulders was chosen based on previous studies on SWE of the rotator cuff, where effects were noted in sample sizes >50.^3,8-11,16,19^ All 115 shoulders completed the 6-week postoperative ultrasound and SWE assessment. There were no losses to follow-up and no missing data for any primary variable.

### Patient Characteristics

The study cohort consisted of 115 shoulders (110 patients, 5 bilateral cases). There were 65 (59%) male and 45 (41%) female patients, with a mean age of 60 years (range, 30-82 years). A total of 81 patients identified a traumatic event that initiated their symptoms. The mean duration of symptoms was 14 months (range, 0-240 months). There was a similar number of full-thickness (n = 58) and partial-thickness (n = 57) tears, with a mean tear size of 2.5 cm^2^ (range, 0.1-8.4 cm^2^) on ultrasound assessment.

### Association Between Preoperative and 6-Week Postoperative SWE of the Supraspinatus Tendon

The mean (± standard deviation) SWE velocity of the supraspinatus tendon decreased by 12% from preoperatively (6 ± 1.8 m/s; range, 2-11 m/s) to 6 weeks after arthroscopic primary rotator cuff repair (5 ± 0.85 m/s; range, 2-8 m/s). The mean SWE elasticity similarly decreased by 30% from 108 ± 70 kPa to 75 ± 27 kPa. The supraspinatus tendons, on average, became elastographically softer in the early postoperative period.

**Figure 3.**
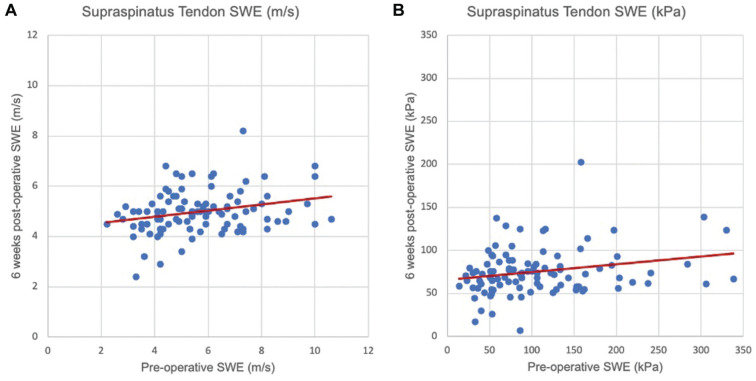
Relationship between preoperative and 6-week postoperative supraspinatus tendon elastographic stiffness. (A) Elastographic stiffness of the repaired supraspinatus tendon in velocity (m/s) (Spearman rho correlation *R* = 0.3; *P* = .01 [95% CI, 0.05-0.43]). (B) Elastographic stiffness of the repaired supraspinatus tendon in elasticity (kPa) (Spearman rho correlation *R* = 0.2; *P* = .04 [95% CI, 0.003-0.39]). SWE, shear wave elastography.

There was a statistically significant but weak positive correlation between the elastographic stiffness of the supraspinatus tendon before and 6 weeks after arthroscopic primary repair (*R* = 0.3, *P* = .01 [95% CI, 0.05-0.43] for velocity [Figure 3A]; *R* = 0.2, *P* = .04 [95% CI, 0.003-0.39] for elasticity [Figure 3B]), indicating partial preservation of relative tendon elastographic stiffness in the early postoperative period. In general, tendons that were elastographically soft preoperatively remained relatively softer at 6 weeks after surgery, and those that were elastographically stiff preoperatively remained relatively stiffer.

### Association Between Preoperative SWE of the Supraspinatus Tendon and Patient and Tear Characteristics

**Figure 4.**
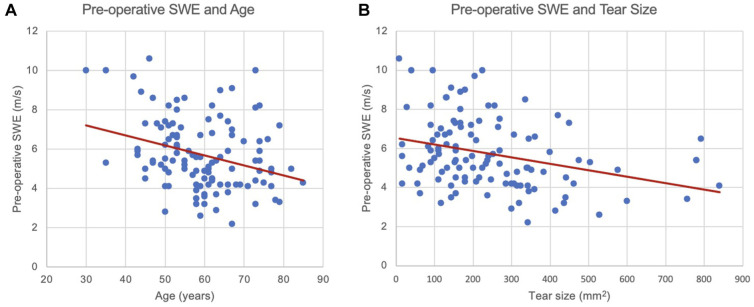
The effect of (A) patient age (Spearman rho correlation *R* = −0.3; *P* = .002 [95% CI, −0.46 to −0.10]) and (B) tear size (Spearman rho correlation *R* = −0.3; *P* < .0001 [95% CI, −0.49 to −0.15]) on the preoperative elastographic stiffness of torn supraspinatus tendons before surgery. SWE, shear wave elastography.

The preoperative elastographic stiffness of the torn supraspinatus tendon correlated negatively with age (*R* = −0.3; *P* = .002 [95% CI, −0.46 to −0.10]) (Figure 4A), tear thickness (*R* = −0.4; *P* = .0001 [95% CI, −0.58 to −0.27]), and tear size (*R* = −0.3; *P* < .0001 [95% CI, −0.49 to −0.15]) (Figure 4B). No association was observed between preoperative SWE and duration of symptoms (*R* = 0.03; *P* = .8).

In other words, the edges of torn supraspinatus tendons were elastographically softer in older patients, in larger tears, and in full-thickness tears, while the edges of torn supraspinatus tendons were elastographically stiffer in younger patients, in smaller tears, and in partial-thickness tears. The preoperative elastographic stiffness of the torn supraspinatus tendon decreased with increasing age, tear thickness, and tear size. Larger tears and tears involving a greater proportion of tendon thickness (full-thickness or high-grade partial-thickness tears) were associated with reduced preoperative tendon elastographic stiffness.

### Predictive Ability of Age, Duration of Symptoms, Tear Thickness, Tear Size, and Preoperative SWE to Determine the Change in Elastographic Stiffness of the Supraspinatus Tendon After Surgical Repair

**Figure 5.**
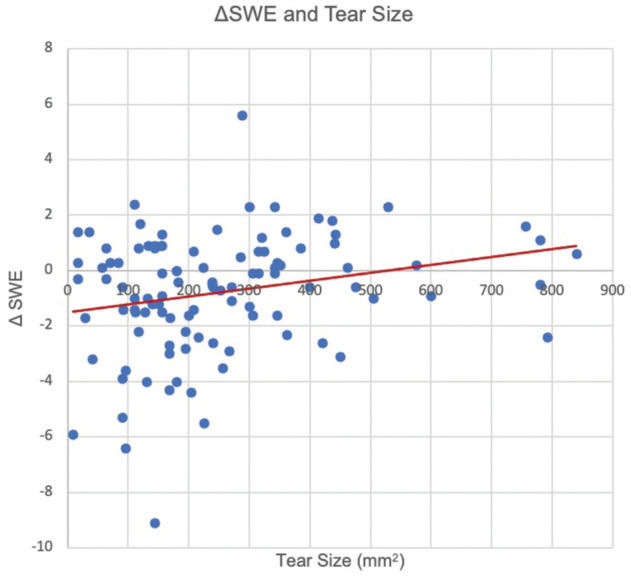
The relationship between the change in shear wave elastography (ΔSWE) of the supraspinatus tendon from before surgery to 6 weeks after surgery (ΔSWE = 6-week postoperative SWE − preoperative SWE) and tear size (Spearman rho correlation *R* = 0.2, *P* = .02 [95% CI, 0.02-0.39]; analysis of variance *F* = 6, *P* = .02, β = .003, SE = 0.001 [95% CI, 0.0005-0.005]).

**Figure 6.**
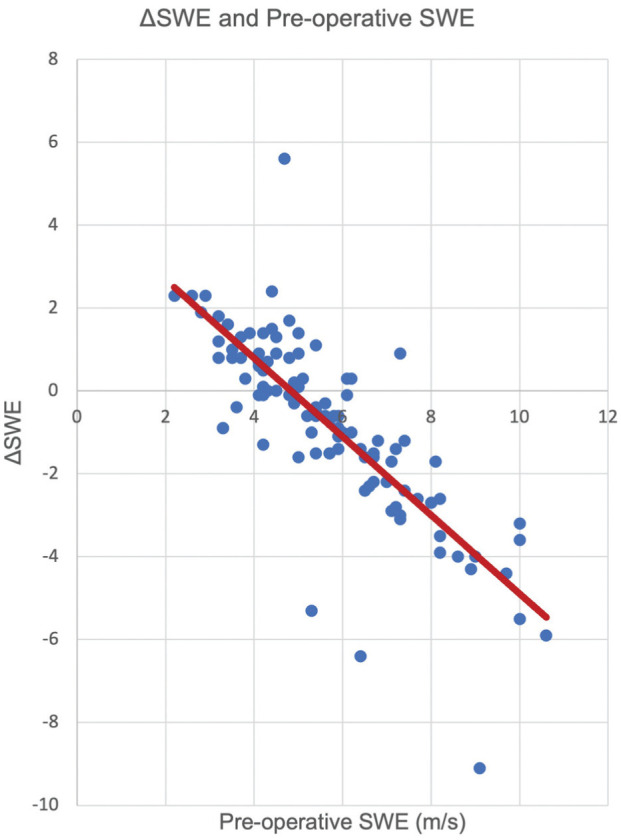
The relationship between the change in shear wave elastography (ΔSWE) of the supraspinatus tendon from before surgery to 6 weeks after surgery (ΔSWE = 6-week postoperative SWE − preoperative SWE) and preoperative elastographic stiffness of the torn supraspinatus tendon (Spearman rho correlation *R* = −0.8, *P* < .0001 [95% CI, −0.8 to −0.76]; analysis of variance *F* = 182, *P* < .0001, β = −0.95, SE = 0.07 [95% CI, −1.09 to −0.81]).

ΔSWE was defined as the difference between SWE measurements before and 6 weeks after arthroscopic primary repair. This represents the change in the elastographic stiffness between the torn supraspinatus tendon and the repaired supraspinatus tendon. In our cohort, the mean ΔSWE from before surgery to 6 weeks after surgery was −0.79 m/s, reflecting an overall early reduction in tendon elastographic stiffness after repair. ΔSWE correlated with tear thickness (*R* = 0.4; *P* < .0001 [95% CI, 0.19 to 0.53]), tear size (*R* = 0.2; *P* = .02 [95% CI, 0.02 to 0.39]) (Figure 5), and very strongly with preoperative SWE (*R* = −0.8; *P* < .0001 [95% CI, −0.8 to −0.76]) (Figure 6). No significant association was observed between ΔSWE and age (*R* = 0.2; *P* = .11).

The strong inverse relationship between preoperative SWE and ΔSWE indicates that tendons with higher initial elastographic stiffness tended to show greater early decreases in stiffness, while those with lower initial elastographic stiffness tended to show smaller decreases or relative increases. This pattern is consistent with regression to the mean, whereby extreme preoperative values move toward the cohort mean at early follow-up. However, the persistence of significant associations between ΔSWE and tear thickness and tear size suggests that this phenomenon is not purely statistical and is also influenced by underlying tendon and tear characteristics.

On univariate regression analysis, tear thickness (*F* = 12; *P* = .001; β = 2.8; SE = 0.8 [95% CI, 1.15 to 4.38]), tear size (*F* = 6; *P* = .02; β = .003; SE = 0.001 [95% CI, 0.0005 to 0.005]) (Figure 5), and preoperative SWE (*F* = 182; *P* < .0001; β = −0.95; SE = 0.07 [95% CI, −1.09 to −0.81]) (Figure 6) were significant predictors of ΔSWE. Interestingly, age was not a significant independent predictor of ΔSWE (*F* = 2; *P* = .2).

On multivariate regression analysis, preoperative SWE emerged as the dominant independent predictor of ΔSWE (*F* = 60; *P* < .0001; *R*^2^ = 0.64; β = .96), with tear thickness (β = .02) and tear size (β = .0003) contributing to a lesser extent. Supraspinatus tendons that were elastographically softer preoperatively, particularly in older patients with larger and more extensive tears, demonstrated significant early improvement in elastographic stiffness after arthroscopic primary repair.

Since ΔSWE was mathematically defined using preoperative SWE, the Oldham method was performed to correct for potential coupling. The resulting scatterplot demonstrated data points scattered randomly around the horizontal line with a very minimal slope of *y* = 3.41^−17^+ 1.85^16^*x*. This indicates that there was no proportional bias and that the relationships we described between preoperative SWE and ΔSWE were not due to statistical coupling and more likely reflect a true biological effect.

## Discussion

The major findings of this study were as follows: (1) there was a modest association between the preoperative and 6-week postoperative elastographic stiffness of the supraspinatus tendon after arthroscopic primary repair; (2) preoperative tendon elastographic stiffness was related to age, tear thickness, and tear size, but not with duration of symptoms; (3) the supraspinatus tendons of older patients and those with larger full-thickness tears were elastographically softer preoperatively than those of younger patients with smaller partial-thickness tears; (4) the potential for improvement or smaller decrease in tendon elastographic stiffness after repair was observed in tendons with lower preoperative elastographic stiffness; and (5) preoperative tendon elastographic stiffness was the strongest independent predictor of early change in postoperative tendon elastographic stiffness among the variables examined.

An unexpected finding was the presence of a regression-toward-the-mean pattern in the elastographic stiffness of the supraspinatus tendon after repair. Tendons that were elastographically softer preoperatively exhibited increases in elastographic stiffness at 6 weeks postoperatively, whereas tendons that were elastographically stiffer preoperatively demonstrated smaller increases or relative reductions in stiffness over the same period. As a result, the range of SWE values was narrower 6 weeks postoperatively compared to the wider range of SWE values evident preoperatively. This regression-toward-the-mean effect provides one explanation for the modest correlation observed between preoperative and 6-week postoperative tendon elastographic stiffness.

A strong inverse relationship was noted between preoperative SWE and ΔSWE. While this association between baseline preoperative SWE values and change scores could have been attributed to mathematical coupling, the results of statistical adjustments to account for this showed no proportional bias, which supports a biological, rather than statistical, effect.

The finding that supraspinatus tendons in older patients and in larger tears were elastographically softer is consistent with previous studies that have examined postoperative SWE measurements. These previous studies have similarly reported lower tendon elastographic stiffness in association with increasing age, tear thickness, and tear size.^6,8,16^

In contrast to our hypothesis, lower preoperative tendon elastographic stiffness was associated with a greater early increase or smaller decrease in tendon elastographic stiffness after repair. Tendons that were elastographically stiffer preoperatively, on the other hand, had smaller increases or relative reductions in stiffness at 6 weeks. In general, though, tendons that were elastographically softer preoperatively remained relatively softer than the tendons that were elastographically stiffer preoperatively, 6 weeks after surgery. Preoperative SWE was the strongest independent predictor of this early change in tendon elastographic stiffness, exceeding the predictive value of age, tear thickness, and tear size.

An early reduction in elastographic stiffness after repair was observed, consistent with our prior postoperative longitudinal SWE studies of the supraspinatus tendon.^8,16^ This reduction in elastographic stiffness has been described to occur during the early postoperative period, from 1 to 6 weeks, and was followed by recovery of tendon elastographic stiffness thereafter, from 6 to 12 weeks.^8,10^ The early postoperative period, from 0 to 12 weeks, corresponds to the time frame during which the repaired tendon is considered most vulnerable to structural failure.

SWE and ΔSWE provide objective measures for evaluating tendon quality. While older patients with large tears have softer tendons,^2,13,14^ and repairs in these tendons are more at risk for suture pull-through,^4^ we observed that these softer tendons have the capacity to become elastographically stiffer 6 weeks after repair. Our findings suggest that tendons that were elastographically softer preoperatively demonstrated larger early changes in SWE after repair; however, the extent to which these changes represent true biological healing or whether these tendons will heal or retear remains uncertain.

Several limitations should be acknowledged in this study. SWE measurements were obtained at a single early postoperative time point, and longer-term follow-up with additional longitudinal time points is required to determine whether early changes in tendon elastographic stiffness are associated with structural integrity or clinical patient-reported outcomes. All ultrasound and SWE measurements were performed by a single experienced musculoskeletal sonographer using a standardized protocol. While this approach minimized interoperator variability, it may limit the generalizability of our findings to other operators. Separate intra- or interobserver reliability was not tested in this study; however, the reliability and validity for this protocol have been established in previous publications.^8,17,19^ The cohort was limited to tears <3 cm, restricting generalizability to larger tears. Finally, the observational design of the study limits causal inference.

Despite these limitations, this study has several methodological strengths. It was conducted as a prospective cohort study with a relatively large sample size. All surgical procedures were performed by a single surgeon using a consistent arthroscopic repair technique, minimizing variability related to surgical method. Preoperative and postoperative SWE measurements were obtained at predefined anatomic locations using reproducible imaging planes, allowing direct comparison of tendon elastographic stiffness before and after repair. This study directly examined the relationship between preoperative elastographic stiffness of the torn supraspinatus tendon and early postoperative stiffness of the repaired tendon, which has not been previously reported.

## Conclusion

SWE, a relatively novel, noninvasive method to measure the stiffness of structures, was utilized to measure the quality of torn supraspinatus tendons before arthroscopic repair in a cohort of patients with rotator cuff tears. Preoperative elastographic stiffness of the supraspinatus tendon measured by SWE was modestly associated with its elastographic stiffness 6 weeks postoperatively. Six weeks after arthroscopic primary repair, a regression-toward-the-mean pattern was observed whereby preoperatively softer tendons exhibited early increases in elastographic stiffness, while preoperatively stiffer tendons exhibited early reductions in elastographic stiffness. Tendons in older patients and tendons with large full-thickness tears had significantly lower preoperative SWE values compared to those in younger patients and those with small partial-thickness tears, similar to what has been described in postoperative tendons. Preoperative SWE was the strongest independent predictor of early change in elastographic stiffness, exceeding age and tear characteristics. This study supports the hypothesis that although elastographically soft tendons are the most likely to retear, they also have the greatest capacity to regain elastographic stiffness after being repaired.
